# Dissecting Out the Molecular Mechanism of Insecticidal Activity of Ostreolysin A6/Pleurotolysin B Complexes on Western Corn Rootworm

**DOI:** 10.3390/toxins13070455

**Published:** 2021-06-29

**Authors:** Matej Milijaš Jotić, Anastasija Panevska, Ioan Iacovache, Rok Kostanjšek, Martina Mravinec, Matej Skočaj, Benoît Zuber, Ana Pavšič, Jaka Razinger, Špela Modic, Francesco Trenti, Graziano Guella, Kristina Sepčić

**Affiliations:** 1 Department of Biology, Biotechnical Faculty, University of Ljubljana, 1000 Ljubljana, Slovenia; matej.jotic@gmail.com (M.M.J.); anastasija.panevska@bf.uni-lj.si (A.P.); rok.kostanjsek@bf.uni-lj.si (R.K.); martina.mravinec@bf.uni-lj.si (M.M.); matej.skocaj@bf.uni-lj.si (M.S.); pavsic25@gmail.com (A.P.); 2 Institute of Anatomy, University of Bern, 3012 Bern, Switzerland; ioan.iacovache@ana.unibe.ch (I.I.); benoit.zuber@ana.unibe.ch (B.Z.); 3 Agricultural Institute of Slovenia, 1000 Ljubljana, Slovenia; jaka.razinger@kis.si (J.R.); spela.modic@kis.si (Š.M.); 4 Bioorganic Chemistry Laboratory, Department of Physics, University of Trento, 38123 Trento, Italy; f.trenti@unitn.it (F.T.); graziano.guella@unitn.it (G.G.)

**Keywords:** aegerolysin, bioinsecticide, MACPF-protein, oyster mushroom, pore-forming protein, western corn rootworm

## Abstract

Ostreolysin A6 (OlyA6) is a protein produced by the oyster mushroom (*Pleurotus ostreatus*). It binds to membrane sphingomyelin/cholesterol domains, and together with its protein partner, pleurotolysin B (PlyB), it forms 13-meric transmembrane pore complexes. Further, OlyA6 binds 1000 times more strongly to the insect-specific membrane sphingolipid, ceramide phosphoethanolamine (CPE). In concert with PlyB, OlyA6 has potent and selective insecticidal activity against the western corn rootworm. We analysed the histological alterations of the midgut wall columnar epithelium of western corn rootworm larvae fed with OlyA6/PlyB, which showed vacuolisation of the cell cytoplasm, swelling of the apical cell surface into the gut lumen, and delamination of the basal lamina underlying the epithelium. Additionally, cryo-electron microscopy was used to explore the membrane interactions of the OlyA6/PlyB complex using lipid vesicles composed of artificial lipids containing CPE, and western corn rootworm brush border membrane vesicles. Multimeric transmembrane pores were formed in both vesicle preparations, similar to those described for sphingomyelin/cholesterol membranes. These results strongly suggest that the molecular mechanism of insecticidal action of OlyA6/PlyB arises from specific interactions of OlyA6 with CPE, and the consequent formation of transmembrane pores in the insect midgut.

## 1. Introduction

The western corn rootworm (WCR; *Diabrotica virgifera virgifera*) causes annual economic losses of over 1 billion dollars in the USA [1,2]. This coleopteran plant pest is generally controlled using chemical insecticides, although these can accumulate in the environment and have harmful effects to non-target organisms, including humans [3,4,5,6]. In addition, transgenic corn hybrids that express proteinaceous crystal toxins (Cry toxins) from the bacterium *Bacillus thuringiensis* (Bt maize) are used to control the WCR [7,8,9]. Cry toxins have insecticidal effects through binding to a variety of protein receptors in the insect midgut epithelium [10], which results in cell perforation and death of the insect.

Insects continuously develop resistance to Bt maize through different mechanisms. Resistance to Cry toxins can occur at any stage of the toxic pathway, but it is most commonly linked to the mutation of the protein receptor gene or the development of regulatory mechanisms that disrupt the production of the toxin receptors [11,12]. This development of resistance against Bt maize by the WCR has recently promoted the search for alternative biopesticides and approaches to combat this pest. Hence, several new bioinsecticides of bacterial origin have been proposed recently [13,14,15,16,17].

Similar to bacteria, mushrooms are organisms that live in highly competitive environments and have therefore developed defence mechanisms against several natural enemies, including nematodes, mites, and insect larvae. These defence molecules also include proteinaceous compounds [18,19,20,21], which might represent new candidates in the search for alternative biopesticides. In this regard, some small (13–20 kDa) lipid-binding mushroom proteins have recently gained significant interest. These belong to the aegerolysin family (Pfam 06355; InterPro IPR009413), and they are produced by oyster mushrooms (genus *Pleurotus*): ostreolysin A6 (OlyA6) from *P. ostreatus*; pleurotolysin A2 (PlyA2) from *P. eryngii*; and erylysin A (EryA) also from *P. eryngii* [22,23,24,25,26,27,28]. These proteins can form complexes with pleurotolysin B (PlyB), their 59-kDa protein partner that has a membrane-attack-complex/perforin (MACPF) domain and is produced by *P. ostreatus*. In this way, the OlyA6/PlyB, PlyA2/PlyB, and EryA/PlyB protein complexes can act as potent and species-specific bioinsecticides for use against selected coleopteran pests such as the WCR and the Colorado potato beetle (*Leptinotarsa decemlineata*) [25]. The toxicities of the three protein complexes against these pests are comparable to (and for Colorado potato beetle, greater than) those of other WCR-specific insecticidal Cry proteins, such as Cry34Ab1/Cry35Ab1 [25,26,29]. Cry34Ab1 is the only representative of the Cry toxin family that is structurally related to the aegerolysins [30].

The activity of these *Pleurotus* aegerolysin/PlyB complexes was suggested to arise from aegerolysin recognition and binding to membrane domains enriched in cholesterol and certain sphingolipids, as sphingomyelin and ceramide phosphoethanolamine (CPE), but not to other membrane lipids [24,25,26,27,28,31,32]. The structure of the PlyA/PlyB complexes in artificial sphingomyelin/cholesterol membranes was resolved by a combination of X-ray crystallography and cryo-electron microscopy (cryo-EM). These showed that PlyA/PlyB assembles into a 13-mer pore of 80 Å in diameter and 100 Å in depth. In each pore subunit, a PlyB molecule is positioned on top of a membrane-bound dimer of PlyA, and the transmembrane regions of PlyB assemble into β-hairpins, to form the membrane-inserted β-barrel [31].

As well as interacting with membranes that contain sphingomyelin, the *Pleurotus* aegerolysins can interact 1000-fold more strongly with artificial lipid vesicles that contain equimolar amounts of cholesterol and CPE [27]. Of note, CPE is the major sphingolipid in invertebrate cell membranes, while it is not found in other taxa [33]. Furthermore, *Pleurotus* aegerolysin-based cytolytic complexes can efficiently bind to and permeabilise artificial lipid vesicles and biological membranes that contain physiologically relevant concentrations of CPE (1–5 mol%) [25,32]. This interaction has been suggested to be responsible for the insecticidal effects of these *Pleurotus* aegerolysins [25].

Investigation of the sphingolipid specificities of the different *Pleurotus* aegerolysins has shown that OlyA6 and PlyA2 can interact with both sphingomyelin/cholesterol and CPE/cholesterol lipid complexes, while EryA, which is an aegerolysin produced by *P. eryngii* and that has 79% amino acid sequence identity with OlyA6, exclusively recognizes the CPE/cholesterol combination [25,27]. Moreover, it has been shown that native EryA has membrane-permeabilisation activity in combination with a MACPF partner protein erylysin B (EryB), which is also produced by *P. eryngii* and has 96% amino acid sequence identity with PlyB [34].

The identification of the receptor(s) responsible for the binding of these insecticidal proteins is very important to form an understanding of the potential for cross-resistance of new technologies that are aimed at maintaining the durability of WCR resistance traits. In the present study, we analysed the histological alterations in the midgut of WCR larvae fed with the insecticidal OlyA6/PlyB mixture. Following our previous studies using surface plasmon resonance [25], we used cryo-EM here to further explore the mechanism of the membrane interactions of the OlyA6/PlyB complex with: (i) lipid vesicles composed of artificial lipids and containing physiologically relevant CPE levels; (ii) lipid vesicles reconstituted from a WCR non-polar lipid extract; and (iii) WCR brush border membrane vesicles. In addition, we analysed the membrane binding and permeabilisation seen for OlyA6 and EryA in combination with the EryA endogenous pore-forming partner protein, EryB [34].

## 2. Results

### 2.1. Lipidomic Analysis of Western Corn Rootworm Larvae

**Figure 1:**
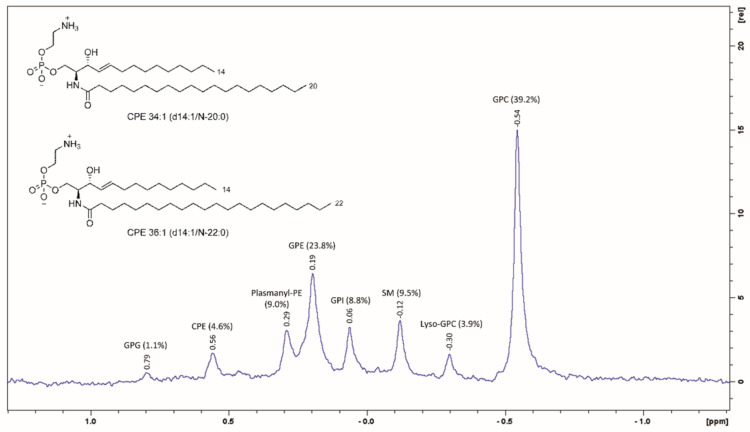
^31^P NMR (162 MHz) spectrum of WCR larvae lipid extract in MeOH-d_4_ at 300 K. The structures of the main two ceramide phosphoethanolamine (CPE) species are shown, which account for 66% of the total CPE.

The non-polar lipid extract from the WCR larvae was analysed by NMR and MS techniques to define the exact lipid composition of the cell membranes. In particular, the relative molar fractions were determined for the phospholipid classes through the ^31^P NMR spectra (Figure 1). Among the phospholipids, the dominant classes were the glycerol-based GPC (peak area integration, 39.2%), GPE (23.8%), plasmanyl-PE (9.0%), and GPI (8.8%), followed by minor amounts of lyso-GPC (3.9%) and glycerolphosphoglycerol lipids (1.1%). Interestingly, the ceramide-based lipids showed relatively high abundance of the phosphocholine sphingomyelin (9.5%) and CPE (4.6%) species. All of these ^31^P NMR signals were assigned by comparisons to our previous NMR measurements carried out on commercially available lipid standards. The sphingomyelin:CPE relative molar ratio was estimated as ~1.7 by peak area integration, which indicated a preference for ceramide linked to the phosphocholine moiety (sphingomyelin) with respect to ceramide linked to the phosphoethanolamine moiety (CPE).

The ^1^H NMR analysis performed on the same WCR lipid extract confirmed the large dominance of GPC and/or the sphingomyelin lipid species (diagnostic peak at δ_H_ 3.22, singlet 9H, for the –N(CH_3_)_3_^+^ group) and of GPE and/or the CPE lipid species (diagnostic peak at δ_H_ 3.16, multiplet 2H, for the –CH_2_-NH_2_ group), over the other phospholipid classes (Figure S1). The relative peak area integration defined a (GPC plus sphingomyelin):(GPE plus CPE) relative molar ratio of 1.59, which was in good agreement with that obtained by area integration of the corresponding ^31^P signals (1.7). ^1^H NMR also revealed a low relative molar ratio of neutral lipids:total phospholipids, with cholesterol (14 mol%) in the upfield region 0.72 > δ_H_ > 1.01 ppm, and triglycerides (17 mol%) in the downfield region 4.45 < δ_H_ < 3.90 ppm (Figure S1).

More comprehensive information on the lipid molecular species in terms of intra-class distributions for different chain lengths and saturation was obtained using HPLC–electrospray ionisation–tandem MS (MS/MS). This analysis led to the identification of the following lipids species: lyso-GPCs (n = 6), GPCs (21), sphingomyelins (9), GPEs (18), GPIs (9), and CPEs (13), as well as neutral triglycerides (TAGs, 29) and diglycerides (DAGs, 9), and free fatty acids (13). The compositions of the DAG, TAG, and lyso-GPC alkyl lateral chains were mostly palmitic (C16:0), oleic (C18:1), and linoleic (C18:2). In particular, the GPCs were mostly represented by the polyunsaturated species of GPC 36:4 and GPC 36:3, followed by the diunsaturated GPC 34:2 and GPC 34:1. The lipid profile of the GPEs revealed this class to be composed of up to 35% plasmanyl-PE, while also noting the two diunsaturated GPEs and plasmanyl-PE O-36:2, GPE 34:2, and GPE 36:3. Among the ceramides, sphingomyelin showed the major component of saturated fatty acid lateral chains as mainly N-18:0 and N-16:0, while CPE showed a wider distribution among the monounsaturated lateral chains. A deeper analysis of the CPE species was performed using triple-quadrupole MRM MS measurements, which revealed the absolute concentration of CPE to be ~6 µg/mg in the total lipid extract. Of these, four species represented 80% of the total CPE: CPE 34:1 (38%), CPE 36:1 (28%), CPE 37:1 (7%), and CPE 35:1 (6%). From the MS/MS data, the most represented CPE 34:1 and CPE 36:1, which together represented 66% of the total CPE, were C14-based sphingosines that were N-acylated by the arachidic (d14:1/20:0) or behenic (d14:1/22:0) acyl chains (Figure 1, inset).

### 2.2. Histological Analysis of OlyA6/PlyB Effects on Midgut Epithelium of Western Corn Rootworm Larvae

**Figure 2:**
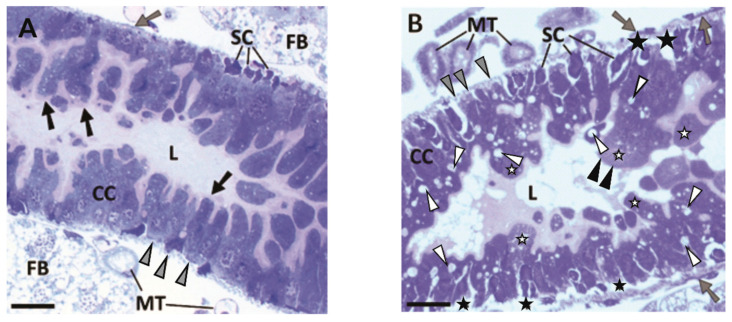
Histopathological changes of midgut and surrounding tissues in WCR larvae. (**A**) Representative section from control group, showing gut epithelium of columnar cells (CC) with continuous layer of microvilli on their apical surface (black arrows) and denser stem cells (SC) at their base. The epithelium is surrounded by a layer of circular (grey arrowheads) and longitudinal (grey arrow) muscle fibres. (**B**) Representative section from OlyA6/PlyB-treated group, showing columnar cells (CC) with swollen apical surface (white stars) protruding into the gut lumen (L), partial absence of microvilli (black arrowheads), and cytoplasmic vacuoles (white arrowheads). Delamination of epithelial cells (black stars) from underlying muscular tissue and formation of pockets surrounding stem cells can also be seen in the basal region of the epithelium (SC). L, gut lumen; FB, fat body; MT, Malpighian tubules. Scale bars: 20 µm.

Microscopy analysis of the midgut and surrounding tissues in the WCR larvae in the control group showed that the gut wall comprised a simple columnar epithelium surrounded by layers of circular and longitudinal muscles. There was a continuous layer of microvilli on the apical surface of the epithelial cells, with smaller stem cells seen at the base of the epithelial cells (Figure 2A). The histopathological effects for the WCR larvae fed on OlyA6 plus PlyB included vacuolisation of the cytoplasm, discontinuation of the microvillus layer, and swelling of the apical surface of the epithelial cells into the gut lumen. Additionally, changes to the basal side of the epithelium included delamination of the columnar cells from the basal lamina and the underlying muscle fibres and formation of ‘pockets’ around the stem cells (Figure 2B). No histopathological effects were seen for the WCR larvae fed with either OlyA6 or PlyB alone (Figure S2).

### 2.3. Cryo-Electron Microscopy of the OlyA6/PlyB Assembly on Large Unilamellar Vesicles and Brush Border Membrane Vesicles

**Figure 3:**
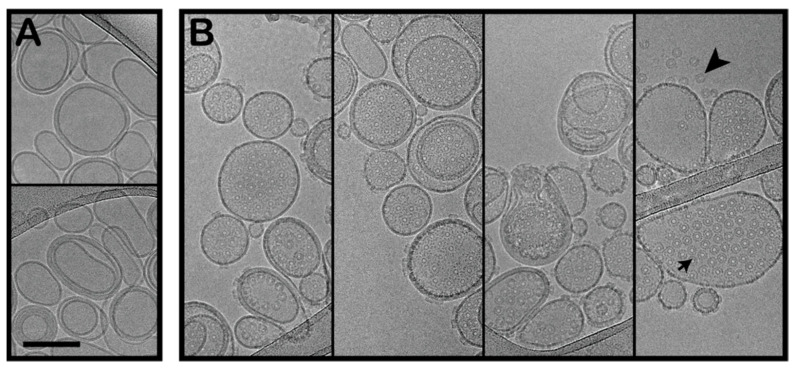
OlyA6/PlyB interaction with CPE-containing membranes. Large unilamellar vesicles (LUVs—0.5 mg/mL; CPE/palmitoyl-oleoyl phosphatidylcholine (POPC)/cholesterol; 5/47.5/47.5; mol/mol/mol) without (**A**) and with (**B**) OlyA6/PlyB added in solution. OlyA6, 3.64 μM; PlyB, 1.67 μM; OlyA6/PlyB, 2.18/1; mol/mol. Arrowhead, detached oligomers in solution; arrow, oligomers in the membranes. Scale bar: 250 nm.

To confirm the binding and pore formation of OlyA6/PlyB heterocomplexes on the CPE-containing membranes, they were directly visualised on the artificial lipid vesicles using cryo-EM. The LUVs containing 5% CPE and incubated with a mixture of 3.64 μM OlyA6 and 1.67 μM PlyB were examined. As previously reported using surface plasmon resonance and calcein release assays [25], there was efficient binding and pore formation of the OlyA6/PlyB oligomers to the artificial membranes containing 5% CPE (Figure 3). Most of the membranes were fully covered with large assemblies of pores, which suggested high-affinity binding and oligomerisation at the lipid to protein ratio used here (233.5/1, mol/mol). As well as the OlyA6/PlyB pores in the LUVs, free OlyA6/PlyB oligomers could be seen in the solution. While oligomerisation and pore formation at the concentrations used for these experiments required the presence of membranes to concentrate the proteins, it is not unusual for the oligomers to be in solution after oligomerisation, which appears to occur after oligomer formation and shedding from the membranes [35,36].

The efficient binding to these CPE-containing artificial membranes prompted us to investigate the formation of OlyA6/PlyB hetero-oligomers on LUVs reconstituted from the non-polar lipids extracted from the WCR larvae. It was not possible to precisely measure the lipid and protein concentrations here to replicate the conditions for the artificial lipids, as the samples were relatively dilute and difficult to screen using cryo-EM. However, oligomers were clearly seen on the membranes of these LUVs reconstituted from the non-polar WCR larva lipids, which confirmed that OlyA6 binding and OlyA6/PlyB hetero-oligomerisation can indeed occur in this system that more closely mimicked the composition of the WCR larvae membranes (Figure S3). OlyA6 assemblies were occasionally seen on membranes (Ioan Iacovache, personal communication).

**Figure 4:**
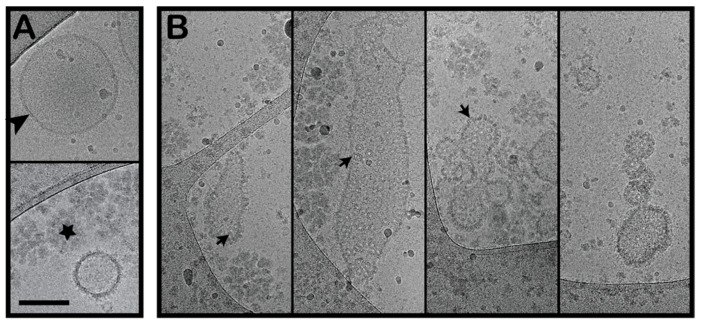
OlyA6/PlyB pores on brush border membrane vesicles (BBMVs) isolated from WCR larvae. BBMVs without (**A**) and with OlyA6/PlyB added (**B**). OlyA6, 13 μM; PlyB, 5.96 μM; OlyA6/PlyB, 2.18/1; mol/mol. Arrowhead, lipid bilayer, and membrane proteins; star, unknown contaminant; arrows, OlyA6/PlyB oligomers. Scale bar: 250 nm.

The LUVs reconstituted from the non-polar WCR larva lipids can be considered as typical lipid membranes in the WCR larvae. As direct contact between the intestinal contents and the cells lining the digestive tract is prevented by the cuticular lining in the foregut and hindgut [37], the apical surface of the midgut epithelium would be the first membrane encountered by ingested toxins in these WCR larvae. Therefore, we investigated the binding of the toxin to membrane vesicles prepared from whole third instar WCR larvae, as the BBMVs. These maintain the lipid composition of the WCR larvae and also retain the membrane proteins and glycolipids [38,39]. These isolated BBMVs were first imaged by cryo-EM to confirm their integrity (Figure 4). While vesicles with the typical lipid bilayer and proteins were clearly seen, these samples also contained an unidentified contaminant, which appeared to be a protein and lipid aggregate. After incubating the BBMVs with OlyA6 and PlyB, the hetero-oligomers were again seen to be inserted into the BBMV membranes. A proportion of the BBMVs also aggregated upon incubation with OlyA6 and PlyB, and these could not be imaged. As well as the OlyA6/PlyB pores in the BBMVs, there were oligomers associated with the aforementioned contaminant fraction.

Taken together, these cryo-EM observations show that OlyA6 and PlyB can indeed efficiently bind and oligomerise on CPE-containing membranes as well as on membranes derived directly from the WCR larvae. Consequently, the OlyA6/PlyB complex forms pores with similar stoichiometry to those observed previously in structural studies of OlyA6/PlyB and PlyA/PlyB binding to membranes that contained sphingomyelin and cholesterol [31,40].

### 2.4. Evaluation of Membrane Binding and Permeabilisation Activity of Erylysin B

**Figure 5:**
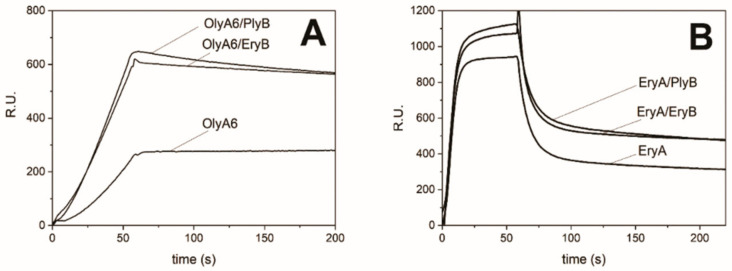
Surface plasmon resonance of the interactions of OlyA6 and EryA with large unilamellar vesicles (LUVs; CPE/POPC/cholesterol; 5/47.5/47.5; mol/mol/mol). LUVs were immobilised (Biacore L1 chip) to approximately 10,000 RU, and analytes injected in vesicle buffer (flow rate, 10 μL/min). Representative sensorgrams of triplicate analyses are shown. Binding to lipid vesicles of 0.5 μM OlyA6 alone or in combination with PlyB or EryB (**A**) and 5 μM EryA alone or in combination with PlyB or EryB (**B**). Aegerolysin/PlyB and aegerolysin/EryB molar ratio, 12.5/1.

Surface plasmon resonance studies with on-chip immobilised LUVs composed of an equimolar palmitoyl–oleoyl phosphatidylcholine (POPC)/cholesterol ratio and supplemented with 5% CPE confirmed the binding of both OlyA6 and EryA to these LUVs. As reported by Panevska et al. [25], EryA showed comparable levels of binding to these LUVs at higher concentrations than those seen for OlyA6, and dissociated more rapidly from these LUVs. Additionally, the interactions of both OlyA6 and EryA with these LUVs were considerably stronger in the presence of either PlyB or EryB. This effect was especially pronounced for OlyA6, where the addition of PlyB or EryB almost doubled the surface plasmon resonance signal compared to the binding of OlyA6 alone (Figure 5).

**Figure 6:**
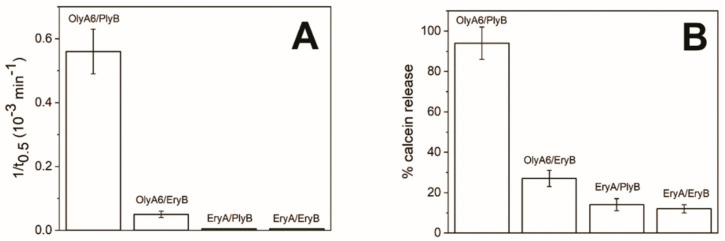
Permeabilisation activity of *Pleurotus* aegerolysins OlyA6 or EryA in combination with MACPF-domain partner proteins (PlyB, EryB). Permeabilisation activity of aegerolysin/MACPF complexes assayed for bovine erythrocytes (**A**) or for calcein-loaded small unilamellar vesicles (CPE/POPC/cholesterol; 5/47.5/47.5; mol/mol/mol) (**B**). Both preparations were exposed to 0.67 μM aegerolysins, alone and combined with MACPF-protein partners (PlyB, EryB). Aegerolysin/MACPF-protein molar ratio, 12.5/1. Data are means ±standard error from three independent biological repetitions.

**Figure 7:**
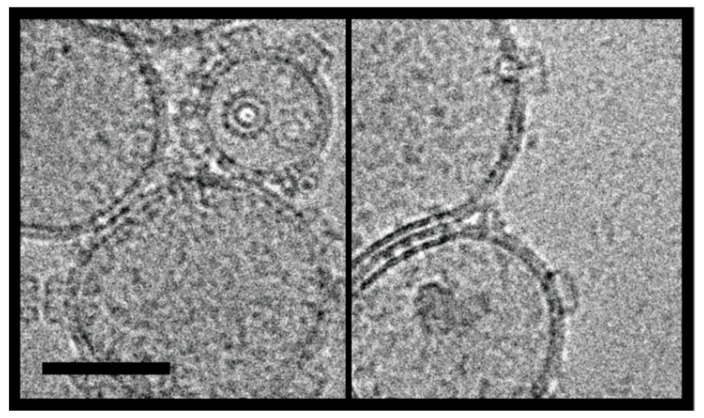
EryA/PlyB pores on large unilamellar vesicles. Cryo-EM of EryA/PlyB pores formed on large unilamellar vesicles (LUVs; CPE/POPC/cholesterol; 5/47.5/47.5; mol/mol/mol). LUVs, 0.5 mg/mL; EryA, 25 μM; PlyB, 2 μM. EryA/PlyB molar ratio, 12.5/1. Scale bar: 100 nm.

In contrast to these surface plasmon resonance studies, in which the addition of both PlyB and EryB induced similar increases in aegerolysin binding to these LUVs, the membrane-permeabilising potentials of aegerolysin/PlyB and aegerolysin/EryB complexes were more diverse. In agreement with the sphingolipid specificities of these aegerolysins, bovine erythrocytes were only lysed by the OlyA6-based cytolytic complexes. The haemolysis was optimal with the naturally occurring MACPF-protein partner of OlyA6, the PlyB, while the haemolysis induced by OlyA6/EryB complexes was lower by ~90% (Figure 6A). The same trend was seen for the effects of the same complexes on calcein-loaded LUVs that contained 5% CPE, where the OlyA6/PlyB and OlyA6/EryB complexes induced the release of 92% and 27% of the calcein, respectively. The permeabilising potential of EryA/PlyB complexes was lower (14%), although this was also comparable to that induced by the EryA/EryB complexes, where EryA was combined with its naturally occurring partner EryB (Figure 6B). These combined binding and permeabilisation studies thus confirm weaker interactions of EryA with CPE-containing membranes, although it is still indicative that the permeabilisation arises from the formation of 13-meric transmembrane pores, as these were clearly seen for EryA and PlyB treatment of these CPE-containing LUVs (Figure 7).

## 3. Discussion

We have shown here that the OlyA6/PlyB insecticidal protein complex from *Pleurotus* mushrooms can form transmembrane pores in the following systems: (i) lipid vesicles made from commercial lipids that contain physiologically relevant concentrations of the insect-specific sphingolipid CPE; (ii) LUVs reconstituted from non-polar lipid extracts from WCR larvae; and (iii) BBMVs from WCR larvae. The oligomeric structures observed for these three different membrane systems are structurally comparable to those formed by OlyA6/PlyB and PlyA/PlyB in sphingomyelin/cholesterol membranes [31,40], with the same stoichiometry. Of note, these data provide the first representations of OlyA6/PlyB pores in membranes containing CPE and also of these pores in insect BBMVs. These systems can explain the mechanism of the insecticidal action of these *Pleurotus* proteins on WCR larvae and also their protective role in nature against such invertebrate predators. The formation of structurally similar transmembrane pores in artificial lipid vesicles containing CPE was also shown here for EryA/PlyB complexes.

OlyA6 is a typical representative of the aegerolysin protein family, which itself consists of approximatively 400 members that are particularly abundant in bacteria and fungi [24,41]. As Cry34Ab1 is the only representative of the *B. thuringiensis* Cry proteins that is structurally related to the aegerolysins [30,42], it is interesting to determine whether the OlyA6-based and Cry34Ab1-based insecticidal complexes act through similar mechanisms.

Cry34Ab1 is part of the bi-component protein complex Cry34Ab1/35Ab1, which has been used in commercial cultivation of transgenic maize in USA since 2005 [42]. The toxicity studies of these Cry34Ab1/35Ab1 protein complexes on several target and non-target organisms are well documented [43], and clearly show their selective toxicity towards the larvae of certain Coleoptera of the family Chrysomelidae, and in particular, the western, southern, and northern corn rootworm [29,42]. The histopathological effects of Cry34Ab1/35Ab1 on the midgut epithelium of WCR larvae have also been studied in detail, which include cell swelling, formation of large vacuoles, enhanced differentiation of stem cells, extrusion of vesicles into the intestinal lumen, collapse of microvilli, and fragmentation of the epithelium [37,44]. For the midgut of the larvae fed on OlyA6/PlyB in the present study, as well as swelling of the apical membrane, there was partial loss of microvilli, vacuolisation of the cytoplasm, and detachment of columnar cells from stem cells and the underlying muscular tissues. These effects most probably arise from the formation of OlyA6/PlyB transmembrane pores, which can be clearly seen in BBMVs obtained from WCR larvae. It is suggestive that this pore formation is the consequence of OlyA6 binding to CPE and/or sphingomyelin in these membranes, with these lipids crucial for effective binding of the aegerolysins to membranes [25,27,32,40] whereby CPE and sphingomyelin represent 4.6 and 9.1 mol% of the phospholipids, respectively, in the WCR membranes.

Similar to the action of OlyA6/PlyB, the swelling and disruption of WCR midgut cells by Cry34Ab1/Cry35Ab1 [37] indicate a membrane-targeted mechanism that might derive from the formation of ion channels. Indeed, it has been shown that bacterial aegerolysin-based insecticidal complexes active against WCR (Cry34Ab1/Cry35Ab1 from *B. thuringiensis* and AfIP-1A/AfIP-1B from *Alcaligenes faecalis*) can permeabilise artificial phospholipid membranes that are devoid of proteins [45,46]. However, this membrane activity appears to be weaker and not as lipid-specific as for the aegerolysins produced by the *Pleurotus* mushrooms [25]. Further, there have been reports of Cry34Ab1 protein receptor(s) in WCR larva midgut membranes [42,47], which appear to be unique and unrelated to other Cry toxins [48]. The binary insecticidal protein complex AflP-1A/AfIP-1B likely shares the same binding sites as Cry34Ab1/Cry35Ab1 in WCR BBMVs [16,46].

To date, we have not been able to show the existence of invertebrate glycan [32] or protein receptors for OlyA6 and the other *Pleurotus* aegerolysins (Panevska and Sepčić, unpublished data). These *Pleurotus* aegerolysins thus appear to exclusively use lipid membrane receptors and to promote their insecticidal effects through the formation of transmembrane pores and colloid-osmotic lysis of the insect midgut cells.

Subtle differences in the primary structures of the otherwise highly similar *Pleurotus* aegerolysins dictate their avidity and selectivity towards specific membrane lipids. All of the *Pleurotus* aegerolysins investigated to date, as well as aegerolysins from some lower fungi (e.g., *Aspergillus niger*) and bacteria (e.g., *Pseudomonas aeruginosa*), recognize and strongly bind to CPE/cholesterol lipid mixtures [25,27,32,49,50]. As well as this binding to CPE as a high-affinity lipid receptor, OlyA6, PlyA2, and PlyA can recognize and bind weakly to sphingomyelin/cholesterol membrane complexes, although EryA does not show this activity [25,27]. Furthermore, the presence of some cholesterol is crucial for the effective membrane activity of OlyA6, which only recognizes the conformation of the cholesterol-associated sphingolipid [25,27,51]. This might explain the higher numbers of OlyA6/PlyB pores that are formed in LUVs that are made of artificial lipids and contain 47.5 mol% cholesterol in comparison with LUVs reconstituted from WCR larva lipids or WCR larva BBMVs, which contain 14 mol% cholesterol.

Similarly, lipidomic analysis of three closely related Chrysomelid beetles (including the Colorado potato beetle) has shown that free sterols and sterol esters account for only about 5 mol% of the total lipidome [52]. The successful pore formation in WCR larva membrane preparations indicates that cholesterol, or some other lipid molecule that acts similarly to cholesterol, induces changes in the sphingolipid conformation that can be recognized by OlyA6.

As for the aegerolysins, the *Pleurotus* fungi produce diverse, but highly similar, MACPF-domain aegerolysin partner proteins, which include PlyB (from *P. ostreatus*) and EryB (from *P. eryngii*). Although EryB is the natural EryA partner in *P. eryngii* [34], the membrane activities of the *Pleurotus* aegerolysins have so far been mainly examined using PlyB. The membrane-binding systems investigated here used different combinations of OlyA6 and EryA with their respective MACPF-protein partners PlyB and EryB, which showed that the membrane binding of both OlyA6 and EryA was considerably stronger in the presence of either PlyB or EryB, which suggests the formation of more stable proteolipid complexes. The membrane permeabilisation studies here confirmed the specific affinity of OlyA6 for its naturally occurring partner, PlyB, due to the greater activity of OlyA6/PlyB complexes than OlyA6/EryB. Interestingly, this was not the case for EryA, which showed comparable membrane-disrupting potential with both of these MACPF-protein partners. These differences might be the result of amino acid differences in the C-terminus of OlyA6 and EryA, as well as differences in the C-terminus of PlyB and EryB, as both of these parts are important for protein–protein interactions and pore formation. Further mutagenesis and structural studies are needed to better explain these findings.

## 4. Conclusions

In conclusion, our combined analyses here strongly suggest that the molecular mechanism of action of these insecticidal aegerolysin-based protein complexes from the *Pleurotus* mushrooms arises from their specific interactions with the invertebrate-specific membrane lipid receptor, CPE. This mode of membrane binding is different from similar aegerolysin-based complexes of bacterial origin (e.g., Cry34Ab1/Cry35Ab1) or other *B. thuringiensis* Cry toxins, as these associate with protein receptors [10,42,47]. As lipids have been conserved across evolution and are constitutive components of cell membranes, the chances that insects can evolve resistance to such aegerolysin-based insecticidal protein complexes through alterations to or degradation of their membrane lipid receptor should be significantly lower.

## 5. Materials and Methods

### 5.1. Materials

All chemicals used in the present study were from Sigma-Aldrich (St. Louis, MO, USA), unless otherwise specified. Ceramide phosphoethanolamine (CPE) was from Matreya LCC (State College, PA, USA), and was dissolved at the final concentration of 5 mg/mL in 1 mL chloroform/methanol (9:1, *v*/*v*) with the addition of 5 μL Milli-Q water. All of the other lipids were from Avanti Polar Lipids (Alabaster, UL, USA), and they were stored at −20 °C and dissolved in chloroform prior to use.

### 5.2. Methods

#### 5.2.1. Protein Purification

The aegerolysins (i.e., OlyA6, EryA, PlyA2) and Δ48PlyB (henceforth, PlyB) were produced as recombinant proteins, as described previously [25,40]. On the basis of the high similarity between Δ48EryB (henceforth, EryB) and PlyB, we used the same protocol for EryB purification as for PlyB. The pET-19b plasmid that contained the EryB gene labelled with C-terminal 6×His-Tag was designed by GenScript Biotech. The plasmid was transformed into Escherichia coli BL21(DE3) competent cells. The cells were then grown in LB Amp medium at 37 °C. When the OD_600_ reached 0.6, 0.5 mM isopropyl-β-D-thiogalactopyranoside was added to the culture to induce protein expression for 4 h at 37 °C. The bacteria were then harvested by centrifugation and lysed by sonication. EryB accumulated in so-called inclusion bodies, which were purified through three different buffers: 20 mM Tris-HCl, pH 8, without additions, plus 0.5 M NaCl and 2% Triton X-100 without and with 2 M urea. The EryB was then dissolved in buffer containing 20 mM Tris-HCl, pH 8.0, 0.5 M NaCl, 6 M guanidine-HCl, 5 mM imidazole, and 5 mM β-mercaptoethanol. Finally, the EryB was purified using an affinity gel (HIS-Select HF Nickel) and dialysed in buffer (20 mM Tris-HCl, pH 8.0, 140 mM NaCl, 2% glycerol).

#### 5.2.2. Rearing of the Western Corn Rootworm and Larva Feeding Tests

Western corn rootworm eggs from the central southeastern European population (i.e., diapause strain) were obtained from the Centre for Agricultural Bioscience International Europe (Switzerland) at the Plant Protection and Soil Conservation Directorate, Hodmezovasarhely, Hungary. The rearing of the WCR larvae was carried out at the Agricultural Institute of Slovenia.

Insecticide tests were performed as previously described [25]. The insect bioassays were performed with neonate larvae over 5 days. The larvae were collected on day 5 after the start of experimental feeding and fixed in 4% formaldehyde in 10 mM phosphate-buffered saline. Approximately 10 larvae per treatment were randomly collected for the histopathological studies.

The treatments were carried out in six-well plates where each well included agar-solidified diet based on SCR artificial diet (Frontier Agricultural Sciences, Newark, DE, USA). The treatments were spread evenly over the entire surface of the diet in the wells using a sterile glass stirring rod, and included exposure to buffer as the negative control (20 mM Tris, pH 8.0, 0.5% glycerol) or to 5.2 µg/cm^2^ OlyA6, 0.4 µg/cm^2^ PlyB, and 5.6 µg/cm^2^ OlyA6/PlyB complex. These doses and duration of exposure were chosen to ensure significant damage to the SCR larva gut tissue based on prior knowledge of the aegerolysin toxicology [25].

#### 5.2.3. Lipid Extraction from Western Corn Rootworm Larvae

Extraction of the total lipids from WCR larvae (at L1+L2+L3 larval stages) was performed using a modified Folch method [53] to separate the polar and non-polar lipid species. Briefly, 0.5 g WCR larvae were added into 1.5 mL ice-cold deionised water with 4 mL of ice-cold methanol. After vigorous vortexing, 2 mL ice-cold chloroform was added, and the suspension was vortexed again. The mixture was extracted overnight on a rotary shaker at room temperature (22 °C) and then centrifuged at 600× *g* for 15 min at 22 °C. The sediment was extracted again and centrifuged as described above. Water was added to the combined supernatants to obtain the final water/methanol/chloroform ratio (5.6:8:4, *v*/*v*/*v*) and then the solution was centrifuged as before to induce separation of the two phases. The upper phase then contained polar lipids, and the lower phase contained the non-polar lipids. After evaporation of the corresponding solvents, the weights of extracted dried lipids were determined, and the polar and non-polar lipids were dissolved in 1 mL water/methanol/chloroform (3:8:4, *v*/*v*/*v*) and methanol/chloroform (1:1, *v*/*v*), respectively.

#### 5.2.4. Lipidomic Analysis of Western Corn Rootworm Larvae

##### Nuclear Magnetic Resonance Analysis

^1^H-NMR (400 MHz) and ^31^P NMR (162 MHz) spectra of the non-polar WCR larva lipid extracts dissolved in MeOH-d_4_ were recorded at 300 K on a nuclear magnetic resonance (NMR) spectrometer (400 MHz; Bruker-Avance, Bremen, Germany), with a 5-mm double-resonance broadband observe (BBO) probe with pulsed-gradient field utility. The ^1^H-90° proton pulse length was 9.3 μs, with transmission power of 0 db. The ^31^P-90° proton pulse length was 17 μs, with transmission power of −3 db. The probe temperature was maintained at 300 K (±0.1 K) using a variable temperature unit (B-VT 1000; Bruker).

Calibration of the chemical shift scale (δ) was carried out on the residual proton signal of the MeOH-d_4_ at δ_H_ 3.310 and δ_C_ 49.00 ppm, and the glycerophosphocholine (GPC) signal at δ_P_ −0.550 ppm was used for calibration of the ^31^P NMR δ scale. The following measurements were performed: ^1^H NMR (i.e., proton chemical shifts, scalar couplings); ^31^P NMR composite pulse decoupling to remove any proton coupling in ^31^P NMR spectra, where generally 4000 free induction decays were acquired and processed using exponential line broadening of 0.3 Hz prior to Fourier transformation; and ^1^H-^1^H COSY (correlation spectroscopy). The resulting 1D NMR spectra were analysed using the MestreNova 12.0 software (Mestrelab Research S.L.2012, Escondido, CA, USA) and TopSpin 3.6.1 (Bruker, Bremen, Germany). The lipid classes from the NMR data were identified through comparisons with our previous NMR measurements carried out on commercially available lipid standards.

##### HPLC–Electrospray Ionisation–Mass Spectrometry Analysis

The lipid extract was analysed by liquid chromatography–mass spectrometry (LC-MS) (Model 1100 series; Hewlett- Packard) coupled to a quadrupole ion-trap mass spectrometer (Esquire LCTM; Bruker, Bremen, Germany) equipped with an electrospray ionisation source and in both positive and negative ion modes.

Chromatographic separation of the phospholipids was carried out at 303 K on a thermostated C18 column (Kinetex TMC18; length, 100 mm; particle size, 2.6 μm; internal diameter, 2.1 mm; pore size, 100 Å; Phenomenex, Torrence, CA, USA). The solvent system consisted of eluant A as MeOH/H_2_O (7:3, *v*/*v*) containing 10 mM ammonium acetate and eluant B as isopropanol/MeOH (10:90, *v*/*v*) containing 10 mM ammonium acetate. Samples were resuspended in 1 mL CHCl_3_/MeOH (2:1, *v*/*v*), and 10 μL was run with a linear gradient from 65% eluant B to 100% B in 40 min, plus 20 min isocratic 100% B at 1 mL/min, to elute the diglycerides and triglycerides. The column was then requilibrated to 65% B for 10 min.

The MS scan range was 13,000 U/s in the range of 50 to 1500 *m*/*z*, with a mass accuracy of ~100 ppm. The nebuliser gas was high purity nitrogen at a pressure of 20 to 30 psi, at a flow rate of 6 L/min and at 300 °C. The electrospray ionisation was operated in positive ion mode for the qualitative and quantitative analyses of GPC, lyso-GPC, and sphingomyelin, and in both positive and negative ion modes for glycerophosphatidylinositol (GPI), glycerophosphatidylethanolamine (GPE), and CPE. For the structural assignments of the lipid species, the extracted ion chromatograms from the positive and/or negative ion full scan data were integrated using the DataAnalysis 3.0 software (Bruker Daltonik, Bremen, Germany). In particular, for GPE and CPE, negative ion mode provided a good yield of molecular ions and information on the length and unsaturation index of the fatty acyl chains, while positive ion mode was used to confirm the correct assignment by analysis of the neutral loss of the polar head. CPE analyses were performed by multiple reaction monitoring (MRM) using a triple quadrupole mass spectrometer (SCIEX API 3000; Applied Biosystems) coupled to a UPLC system (Prominence; Shimatzu). The column, eluents, and gradients were as described above. The calibration curve was calculated using the standard *N*-Acyl-D-erythro-sphingosylphosphorylethanolamine (Matreya).

#### 5.2.5. Preparation of Lipid Vesicles and Brush Border Membrane Vesicles

Multilamellar lipid vesicles were prepared from non-polar lipid extracts from WCR larvae (final lipid concentration, 20 mg/mL) following a previously described procedure [54]. The multilamellar vesicles from commercial lipids (final lipid concentration, 5 mg/mL) were made according to Vezočnik et al. [55]. In both cases, the multilamellar vesicles were suspended in vesicle buffer (20 mM Tris, pH 7.5, 140 mM NaCl, 1 mM EDTA). To prepare large unilamellar vesicles (LUVs), suspensions of multilamellar vesicles were subjected to eight freeze–thaw cycles and then extruded through 0.1-µm-pore-size polycarbonate filters (Millipore, Darmstadt, Germany) at 40 °C. Brush border membrane vesicles (BBMVs) were prepared from whole third instar WCR larvae using the differential magnesium precipitation method [38].

#### 5.2.6. Histological Analysis

After transfer into the fixative (4% formaldehyde, 10 mM phosphate-buffered saline), WCR larvae were exposed to a vacuum by removal of the air above the fixative by syringe until they sank, and then fixed overnight. Fixed specimens were washed in 10 mM phosphate-buffered saline and dehydrated through a graded ethanol series (i.e., 50%, 70%, 90%, 90%, 96%), followed by acetone, and embedded in Agar 100 resin (Agar Scientific, Stansted, UK). Sagittal sections (300 nm thick) of the specimens were stained with Richardson’s stain and observed under a light microscope (Axioimager Z.1; Zeiss, Oberkochen, Germany).

#### 5.2.7. Cryo-Electron Microscopy Sample Preparation and Data Acquisition

The LUVs were mixed with the proteins in the vesicle buffer, with the BBMVs mixed with the proteins in MET buffer (0.3 M mannitol, 5 mM EGTA, 17 mM Tris-HCl, pH 7.5). The LUVs and BBMVs were first incubated with OlyA6 for 30 min at room temperature, then PlyB was added, and the samples were incubated again for 30 min at room temperature. Three microlitres of the samples was applied to a Lacey carbon grid (quantifoil, Cu 200 mesh) after glow discharging (20” 10 mA Baltzers CTA 010). The grid was subsequently mounted and vitrified by plunge-freezing in liquid ethane using a vitrification system (FEI Vitrobot Mark IV). The vitrified sample grids were stored in liquid nitrogen prior to acquisition. Acquisition was on an electron microscope (FEI Tecnai F20) equipped with a direct electron detector (Falcon III) using a side-entry cryo holder (Gatan 626). Image acquisition was performed using the TIA/EPU software with a total dose between 60–80 e^−^/Å^2^. Image processing was then carried out (Fiji/ImageJ) [56,57].

#### 5.2.8. Surface Plasmon Resonance Analysis of Lipid Binding

The aegerolysin/LUV interactions were monitored using a surface plasmon resonance-based refractometer (Biacore X; GE Healthcare, Chicago, IL, USA) and an L1 sensor chip, with vesicle buffer as the running buffer. After the initial cleaning of the chip with regeneration solutions of sodium dodecyl sulphate and octyl β-D-glucopyranoside with 1 min injections at a flow rate of 10 μL/min, the LUVs (0.5 mg/mL) from a mixture of commercial lipids (CPE/POPC/cholesterol 5/47.5/47.5; mol/mol/mol) were bound to the second flow cell of the sensor chip to reach responses of ~10,000 RU at a flow rate of 2 μL/min. The first flow cell was left empty and was used to control for possible non-specific binding of the proteins tested to the chip dextran matrix. The non-specific binding of the proteins was minimised using a 1 min injection of 0.1 mg/mL bovine serum albumin at a flow rate of 30 μL/min. For monitoring the binding on LUVs, OlyA6 (0.5 μM) or EryA (5 µM) were injected for 60 s, followed by a dissociation time of 180 s using the running buffer at a 10 µL/min flow rate. Alternatively, the aegerolysins were injected in combination with their MACPF-protein partners, PlyB or EryB, in the aegerolysin/MACPF-protein molar ratio of 12.5/1. Chip regeneration between injections was with 1 min injections of 0.5% sodium dodecyl sulphate, 40 mM β-D-glucopyranoside, and 30% ethanol, with a flow rate of 10 μL/min. Experiments were performed at 25 °C. The data were processed with the BIAevaluation software (GE Healthcare).

#### 5.2.9. Lipid Vesicles Permeabilisation Assay

Small unilamellar vesicles loaded with calcein at the self-quenching concentration (80 mM) were prepared as described by Sepčić et al. [54]. Vesicle permeabilisation was determined using a fluorescence microplate reader (Tecan, Männedorf, Switzerland) with excitation and emission at 485 and 535 nm, respectively. Calcein-loaded vesicles composed of CPE/POPC/cholesterol (5/47.5/47.5, mol/mol/mol) in vesicle buffer were exposed to 0.67 μM of the different aegerolysins, alone and combined with their MACPF-protein partners, PlyB or EryB, at the aegerolysin/MACPF-protein molar ratio of 12.5/1. The experiments were run in three independent biological replicates for 30 min at 25 °C. The permeabilisation induced by the lytic aegerolysin complexes was expressed as the proportion (%) of the maximal permeabilisation obtained by addition of the detergent Triton-X 100 to a final concentration of 1 mM.

#### 5.2.10. Haemolytic Activity

Haemolytic activity was measured by the turbidimetric method described previously [54]. Typically, 100 μL of the different aegerolysins (concentration range, 0 to 6.7 μM), alone and combined with the MACPF-protein partners, PlyB or EryB, at the aegerolysin/MACPF-protein molar ratio of 12.5/1 in vesicle buffer, was added to 100 μL bovine erythrocyte suspension in vesicle buffer with an apparent absorbance of 0.5 at 650 nm. The decrease in the apparent absorbance was recorded for 30 min at 650 nm using a microplate reader (VIS kinetic; Dynex Technologies, Chantilly, VA, USA) to determine the time necessary for 50% haemolysis, t_0.5_. All of the experiments were performed in triplicates at 25 °C.

## Data Availability

All of the data generated or analysed during this study are included in this published article (and its Supplementary InformationSupplementary Files).

## Supplementary Materials

The following are available online at https://www.mdpi.com/article/10.3390/toxins13070455/s1, Figure S1: 1H NMR spectrum (400 MHz) of WCR larvae lipid extract in MeOH-d4 at 300 K, Figure S2: Histology of midgut and surrounding tissues in WCR larvae treated with OlyA6 (A) and PlyB (B), Figure S3: OlyA6/PlyB pores on large unilamellar vesicles (LUVs) reconstituted from non-polar lipids from WCR larvae.
