# Supplementary material for: Dissecting Out the Molecular Mechanism of Insecticidal Activity of Ostreolysin A6/Pleurotolysin B Complexes on Western Corn Rootworm
Source: Toxins (Basel). 2021 Jun 29;13(7):455. doi: 10.3390/toxins13070455 (PMC8310357; doi:10.3390/toxins13070455)
Supplement: Supplementary file 1 [file toxins-13-00455-s001.zip › toxins-1272464-supp final.pdf]

Supplementary Materials: Dissecting out the molecular mechanism of insecticidal activity of ostreolysin A6/pleurotolysin B complexes on western corn rootworm

Matej Milijaš Jotić, Anastasija Panevska, Ioan Iacovache, Rok Kostanjšek, Martina Mravinec, Matej Skočaj, Benoît Zuber, Ana Pavšič, Jaka Razinger, Špela Modic, Francesco Trenti, Graziano Guella and Kristina Sepčić

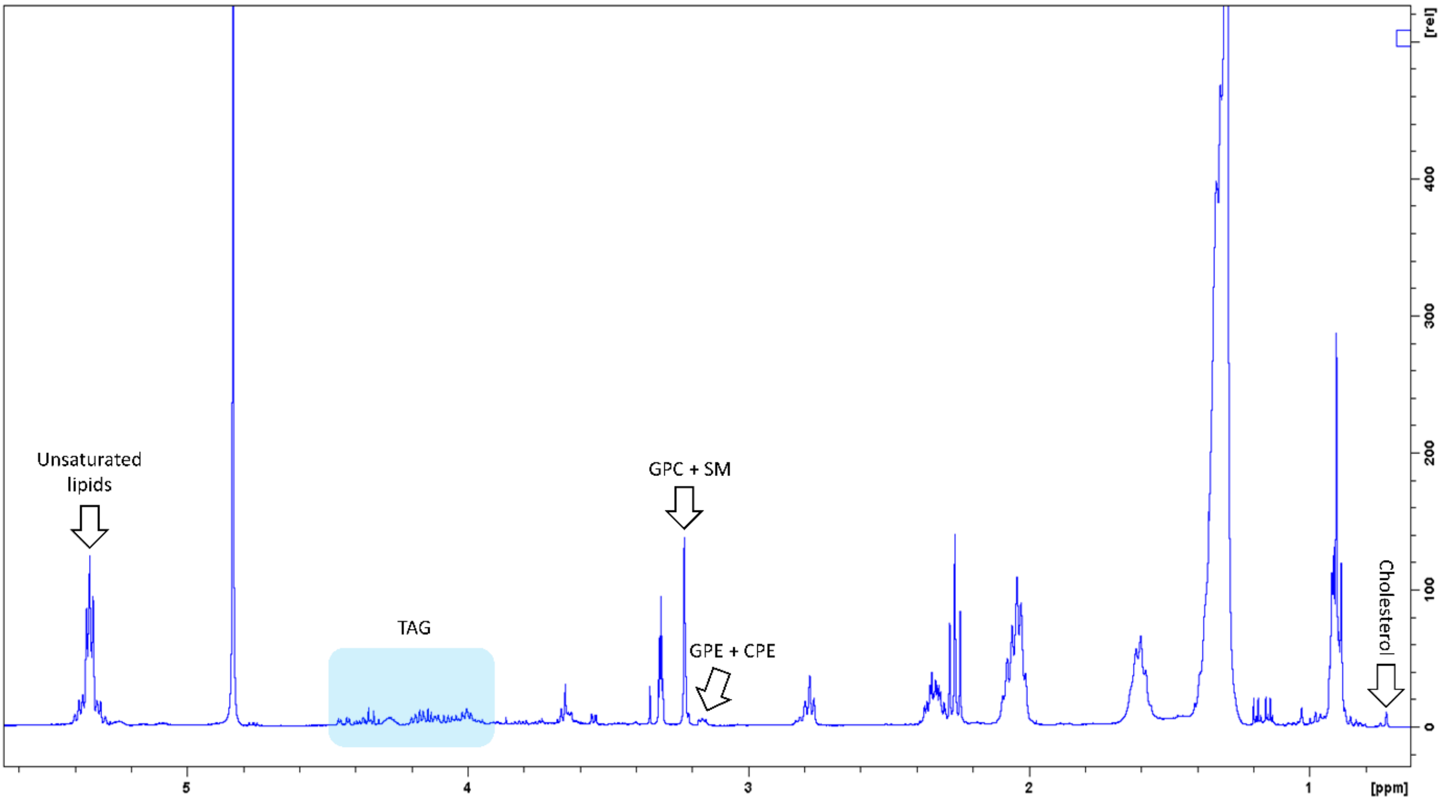

Figure S1. ^{1}H NMR spectrum (400 MHz) of WCR larvae lipid extract in MeOH-d_4 at 300 K.

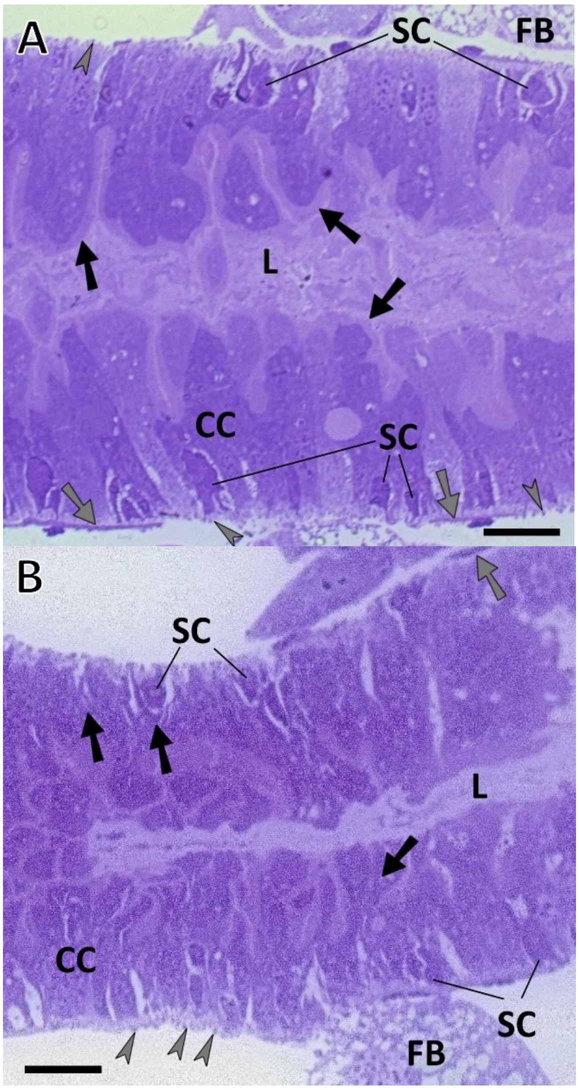

Figure S2. Histology of midgut and surrounding tissues in WCR larvae treated with OlyA6 (A) and PlyB (B). Representative sections for both treatments reveal intact gut epithelium with columnar cells (CC) and continuous layer of microvilli on their apical surface (black arrows), and denser stem cells (SC) at their base. The epithelium is surrounded by a layer of circular (grey arrowheads) and longitudinal (grey arrows) muscle fibres. L, gut lumen; FB, fat body. Scale bars: 20 μm.

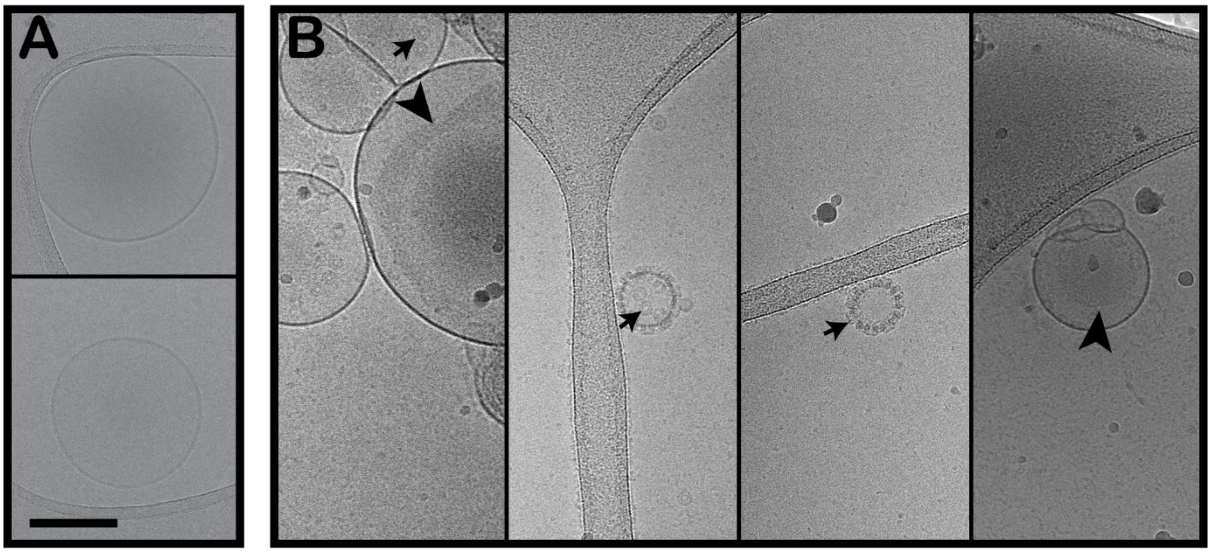

Figure S3. OlyA6/PlyB pores on large unilamellar vesicles (LUVs) reconstituted from non-polar lipids from WCR larvae. LUVs without (**A**) and with OlyA6/PlyB added (**B**). OlyA6, 13 μM; PlyB, 5.96 μM; OlyA6/PlyB, 2.18/1; mol/mol. Arrowheads, OlyA6 membrane-bound aggregates; arrows, OlyA6/PlyB hetero-oligomers. Scale bar: 250 nm.
